# Efferocytosis in the tumor microenvironment

**DOI:** 10.1007/s00281-018-0698-5

**Published:** 2018-09-05

**Authors:** Thomas A. Werfel, Rebecca S. Cook

**Affiliations:** 1 Department of Cell and Developmental Biology, Vanderbilt University School of Medicine, 759 Preston Research Building, 2220 Pierce Ave, Nashville, TN 37232 USA; 2 Program in Cancer Biology, Vanderbilt University School of Medicine, Nashville, TN 37232 USA; 3 Vanderbilt Ingram Cancer Center, Vanderbilt University Medical Center, Nashville, TN 37232 USA; 4 Department of Biomedical Engineering, Vanderbilt University School of Engineering, Nashville, TN 37232 USA

**Keywords:** Efferocytosis, Apoptotic cell death, MerTK, Phosphatidyl serine, macrophage, Tumor microenvironment

## Abstract

Within the course of a single minute, millions of cells in the human body will undergo programmed cell death in response to physiological or pathological cues. The diminished energetic capacity of an apoptotic cell renders the cell incapable of sustaining plasma membrane integrity. Under these circumstances, intracellular contents that might leak into the surrounding tissue microenvironment, a process referred to as secondary necrosis, could induce inflammation and tissue damage. Remarkably, in most cases of physiologically rendered apoptotic cell death, inflammation is avoided because a mechanism to swiftly remove apoptotic cells from the tissue prior to their secondary necrosis becomes activated. This mechanism, referred to as efferocytosis, uses phagocytes to precisely identify and engulf neighboring apoptotic cells. In doing so, efferocytosis mantains tissue homeostasis that would otherwise be disrupted by normal cellular turnover and exacerbated further when the burden of apoptotic cells becomes elevated due to disease or insult. Efferocytosis also supports the resolution of inflammation, restoring tissue homesostasis. The importance of efferocytosis in health and disease underlies the increasing research efforts to understand the mechanisms by which efferocytosis occurs, and how a failure in the efferocytic machinery contributes to diseases, or conversely, how cancers effectively use the existing efferocytic machinery to generate a tumor-tolerant, immunosuppressive tumor microenvironment. We discuss herein the molecular mechanisms of efferocytosis, how the process of efferocytosis might support a tumor ‘wound healing’ phenotype, and efforts to target efferocytosis as an adjunct to existing tumor treatments.

## Introduction

Dying cells are present in most tissues, due to both physiological and pathological cues. Cell death is a key biological process used during developmental stages, when tissues are being formed and shaped. For example, the epidermal webbings that exist between the digits of the developing forelimb of a mammal are removed through the process of apoptosis, forming the articulation of each independent digit [1]. During mammary gland morphogenesis, the ductal epithelium forms initially as a solid cord, but becomes canalized through the process of selective apoptosis of centrally located mammary epithelial cells (MECs) [2]. Even within a fully mature animal, cellular turnover is seen in most tissues, as this process is responsible for removing aged, expired, or unwanted cells. In some circumstances, physiologically induced cell death can occur in a dramatic wave of widespread apoptosis throughout the tissue. For example, the milk-producing MECs that populate the breast during pregnancy and then terminally differentiate during lactation are energetically expensive to maintain, and thus are culled from the breast upon weaning of offspring through the induction of programmed cell death [3]. In rodent models, it is estimated that the widespread cell death characteristic of post-lactational involution removes up to 90% of all MECs in the mammary gland within 7–10 days, representing a powerful model to examine the causes and consequences of cell death within a physiological context. The physiological necessity of apoptosis is illustrated by the developmental anomalies and age-related diseases that occur upon perturbation of apoptosis, including aberrant cell accumulation, cancer, and other pathologies [4]. Pathological cues, such as infection, hypoxia, radiation, or injury similarly can induce widespread cell death in a tissue [4].

Regardless of the causes of apoptosis, or the number of apoptotic cells generated within a tissue, it is remarkable that apoptotic cell death procedes without inducing inflammation, particularly considering what happens to dying cells in the absence of any external influence. Left to its own devices, a dying cell will ultimately lose membrane integrity, leaking cytoplasmic and nuclear contents into the tissue microenvironment [5]. However, it is quite rare under physiological circumstances that an apoptotic cell would lose its membrane integrity and leak its highly inflammatory intracellular contents into tissues, a process referred to as secondary necrosis. The reason for the scarcity of secondary necrosis under physiological conditions, despite the constant burden of apoptotic cells, is that phagocytic cells residing within each tissue, or recruited to the tissue, are adept at recognizing and engulfing apoptotic cells before apoptotic cell membrane integrity is lost. The phagocytic engulfment of apoptotic cells is a process termed efferocytosis [6, 7].

Efferocytosis can be accomplished by professional, non-professional, and specialized phagocytes, and each plays a necessary role in maintaining tissue homeostasis in the event of local cell death. Macrophages and dendritic cells (DCs) are professional phagocytes that are found in most tissues and are the most commonly studied of the efferocytes. Macrophages of the spleen and liver are responsible for clearing expired erythrocytes from the circulating blood [8], migroglia (a specialized central nervous system macrophage) clear dying neurons from the central nervous system [9], and resident lung macrophages are critical for removal of dying alveolar epithelial cells [10]. However, non-professional phagocytes are capable of the important homeostatic role of efferocytosis as well. For example, MECs in the mammary gland are critical for efferocytosis during the earliest stages of post-lactational involution [11, 12]. Similarly, lung airway epithelial cells (LAECs) are capable of ingesting apoptotic neighboring LAECs [10]. Fibroblasts [13], endothelial cells [14], Sertoli cells of the testis [15], and retinal pigment epithelial cells (RPECs) [16] are other known examples of non-professional phagocytes with key roles in sustaining tissue homeostasis and health through efferocytosis.

Loss of efferocytic capabilities within a given tissue can severely impair tissue homeostasis, resulting in inflammation-induced tissue damage and decreased tissue function. For example, mice deficient for MerTK, a receptor tyrosine kinase (RTK) required by MECs for binding to and engulfing apoptotic cells, are incapable of clearing the widespread burden of apoptotic cells from the mammary gland during post-lactation involution, resulting in severe mammary gland inflammation (mastitis), tissue damage, and peri-ductal scarring [12]. MerTK is similarly required by RPECs for engulfment of photoreceptor outer segments that are diurnally released [17]. In the retina, MerTK loss causes inflammation (retinitis pigmentosa), tissue damage and in many cases, age-related blindness [18]. In the testis, MerTK-deficient Sertoli cells are incapable of ingesting the dying germ cells generated during spermatogenesis, predisposing male MerTK-deficient mice to testicular inflammation and decreased fertility [19]. Thus, efferocytosis prevents inflammation in tissues where dying cells are present, in large part by preventing the secondary necrosis of dying cells. Once membrane intergrity is lost, cytosolic and nuclear contents released by the dying cell will inform neighboring cells, particularly macrophages and DCs [20], that danger may be present. Because necrosis is often the result of infection or injury, the released cytoplasmic and nuclear contents represent danger-associated molecular patterns (DAMPs) that induce inflammatory cytokine and chemokine expression from neighboring cells, which rapidly mobilize both innate and adaptive immune responses, in an effort to limit the injurious process at hand [21]. Thus, efferocytosis prevents unwanted inflammation by engulfing the apoptotic cell before secondary necrosis allows leakage of DAMPs into the extracellular space.

However, efferocytosis modulates immunity well beyond the prevention of inflammation. Following engulfment of apoptotic cells, phagocytes are instructed to produce anti-inflammatory cytokines such as transforming growth factor (TGF)-β and interleukin (IL)-10, while actively dampening their expression of pro-inflammatory cytokines such as IL-12 and tumor necrosis factor (TNF) [22]. Immunomodulatory cytokine regulation occurs both in professional and in non-professional phagocytes and is coupled to the efferocytic process. This molecular coupling ensures that efferocytosis is more than ‘immunologically silent,’ but rather actively enforces immune tolerance, wound healing, and homeostasis. Thus, it is not surprising that aberrations in the process of efferocytosis might produce a variety of pathological consequenses, or conversely, that tumors might capitalize from the tolerogenic nature of efferocytosis to support cancer progression and evade immune surveillance. Here, we review the basic mechanisms of efferocytosis, its role in the tumor microenvironment, and potential treatment strategies to combat the pro-tumorogenic effects of efferocytosis in the TME.

### Mechanisms of efferocytosis

**Fig. 1:**
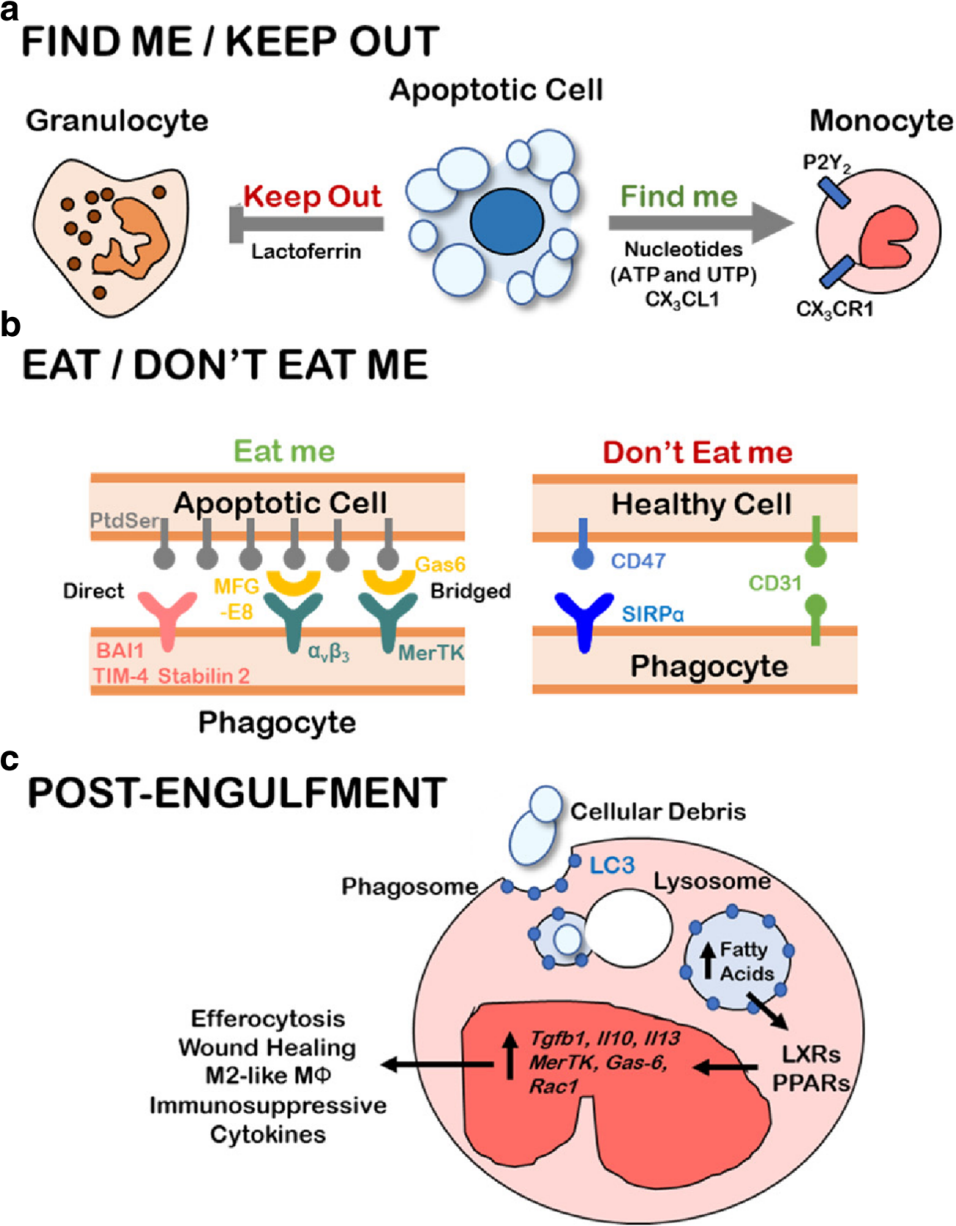
The stages of efferocytosis: Find Me, Eat Me, and Post-Engulfment Signaling. **a** Apoptotic cells release nucleotides (ATP/UTP) and CX_3_CL1 as “Find me” signals that biochemically attract monocytes by engaging the P2Y_2_ and CX_3_CR1 receptors, respectively. Lactoferrin has potent and specific anti-migratory effects on neutrophils and is released by apoptotic cells as a “Keep out” signal for inflammatory cells. **b** Phagocytes must preferentially identify apoptotic cells for efferocytosis while leaving healthy cells unengulfed. Apoptotic cells display PtdSer lipid on the outer leaflet of the cell membrane, which is recognized by phagocytes either directly through BAI1, TIM-4, or Stabilin 2 or indirectly through the αvβ3-MFG-E8 or MerTK-Gas6 bridging combinations. In addition to lower PtdSer outer-leaflet exposure, healthy cells express “Don’t eat me” signals such as CD47 and CD31 which prevent phagocyte activation and engulfment. **c** Apoptotic debris are engulfed via LC3-associated phagosomes which fuse with lysosomes and efficiently degrade cellular contents of the apoptotic cell. The production of fatty acids such as 25-hydroxysterol result in the activation of nuclear receptors and transcriptional regulators, PPAR and LXR, whose activation promotes transcription of pro-efferocytic machinery (MerTK, Gas-6, Rac1) and immunosuppressive cytokines (TGF-β1, IL-10, IL-13)

The process of clearing dying cells from tissue occurs in a series of coordinated and sequential molecular events that eventually result in phagocytosis of the dying cell [23]. Early in the process of apoptosis, the dying cell secretes biochemical mediators, referred to as “find-me” signals, to recruit phagocytes to their proximity (Fig. 1). Find-me signals enable the rapid recognition of dying cells by phagocytes at the earliest stages of apoptosis, which is an important aspect of rapid apoptotic cell clearance. Indeed, efferocytosis can procede even when caspase-mediated apoptosis is not yet fully executed to completion. Four “find-me” signals have been identified thus far: nucleotides (e.g., adenosine triphosphate [ATP]) [24], CX_3_CL1 (chemokine C-X_3_-C motif ligand 1) [25], lysophosphotidylcholine (LPC) [26], and sphingosine 1-phosphate (S1P) [27]. Although evidence is still needed to confirm the activity of LPC and S1P in animal models, nucleotides and CX_3_CL1 both have been shown to recruit monocytes to areas of programmed cell death in vivo. For instance, Elliott et al. showed that triphospohate nucleotides, but not di- and mono-phosphates, attract monocytes in vivo through P2Y_2_ receptor binding when injected into a mouse dorsal airpouch model (Fig. 1a) [24]. Moreover, enzymatic ablation of triphosphate nucleotides resulted in accumulation of cellular corpses, suggesting impaired efferocytosis under triphosphate nucleotide depletion, and illustrating the importance of ‘find-me’ signaling within the efferocytoic pathway.

While “find-me” signals help recruit phagocytes within close proximity of dying cells, “eat-me” signals serve as specific cell surface markers that enable phagocytes to uniquely identify apoptotic cells once the phagocyte arrives on site (Fig. 1b). Once in range, phagocytes recognize the “eat-me” signals displayed on the apoptotic cell. The most heavily studied “eat-me” signal is phosphatidyl serine (PtdSer), a phospholipid normally localized to the inner leaflet of the plasma membrane of healthy cells. Upon initiation of apoptosis, PtdSer is rapidy translocated to the plasma membrane exterior leaflet of the apoptotic cell [28]. It is estimated that PtdSer exposure increases approximately 300-fold within 1–2 h after initiation of apoptosis, flagging the apoptotic cell for engulfment by a neighboring phagocyte [29].

Multiple engulfment receptors expressed by phagocytes are responsible for binding to PtdSer on the dying cell (Fig. 1b). For example, brain angiogenesis inhibitor 1 (BAI1) [30], T cell immunoglobulin and mucin domain containing-4 (TIM-4) [31], and Stabilin-2 [32] bind directly to PtdSer, allowing phagocytes that express BAI-1, TIM-4, or Stabilin-2 to bind dying cells directly. Alternatively, PtdSer can interact with a ‘bridging ligand’ that simultaneously binds to receptors expressed upon a phagocyte. For example, the soluble bridging ligands growth arrest specific-6 (Gas6) [33], Protein S (ProS1) [34], Galectin-3 (Gal3) [35], Tubby and Tubby-like Protein-1 (TULP1) [36] bind to PtdSer on the apoptotic cell and simultaneously bind to the MerTK on the phagocyte. Gas6 also bridges the apoptotic cell to the MerTK-related RTKs Axl and Tyro3, which are expressed by macrophages and DCs [37]. Lactadherin, also known as milk fat globule-EGF factor 8 (MFG-E8) bridges PtdSer of the dying cell to α_v_β_5_ and α_v_β_3_ integrins expressed on the phagocyte [38]. Scavenger receptors, lectins, and complement receptors also serve as PtdSer-binding molecules expressed by phagocytes that aid in recognition of apoptotic cells [39].

However, PtdSer recognition and binding are not sufficient to drive efferocytosis of an apoptotic cell. For example, it is possible for a cell to expose PtdSer on its outer surface without inducing efferocytosis [29]. In certain scenarios, this is due to the need for co-stimulation with second messengers such as calreticulin [40, 41], although the exact mechanisms dictating the need for costimulation remain inconclusive. It is also proposed that, while phagocytes scan for apoptotic cell ‘eat me’ signals, phagocytes simulataneously scan for ‘don’t eat me’ signals (Fig. 1b). Two well-studied ‘don’t eat me’ markers include CD47, a ubiquitously expressed integrin- and thrombospondin-binding protein, and CD31, an integrin-activating protein expressed by hematopoietic lineages. Both CD31 and CD47 inhibit the engulfment of healthy cells that otherwise might express PtdSer on their surface, such as platelets and endothelial cells [42, 43]. This function is particularly important, given that PtdSer, ProtS, and Gas6 each have roles in platelet aggregation and vascular wound repair [44]. Thus, the recognition of apoptotic cells for clearance involves a complex interplay between ‘find me,’ ‘eat me,’ and ‘do not eat me’ signaling.

Upon recognition and tethering of apoptotic cells to the phagocyte, cytoskeletal rearrangement within the phagocyte induce pseudopodia extension to surround the apoptotic cell in what is referred to as the ‘phagocytic cup,’ eventually engulfing the membrane-encapsulated dying cell into what is known as the phagosome. The precise intracellular molecular signaling pathways initating cytoskeletal rearrangements for the formation of the phagocytic cup and the phagosome are still being elucidated. However, it is clear that multiple signaling pathways converge on Rac1, a small GTPase that serves as a potent activator of cytoskeletal dynamics and that is required for efferocytosis [38]. Inhibition of Rac1 signaling impairs engulfment of dying lung epithelial cells by LAECs [45] and blocks engulfment of photorecepter outer segments by RPECs in the retina [46].

Receptors expressed by the phagocyte that bind to the bridging molecules Gas6, ProtS, and MFG-E8 have been shown to engage Rac1 through the guanine nucleotide exchange factor (GEF) complex formed by the two proteins ELMO1 and DOCK180 [47]. However, ELMO/DOCK180 does not bind directly with MerTK, integrin α_v_β_3_, or the other briding molecule receptors. In the case of MerTK, its tyrosine kinase activity results in tyrosine phosphorylation of SH2 and SH3 domains in its cytoplasmic tail, generating binding sites for the adaptor moeclule CrkII, which recruits ELMO/DOCK180 to MerTK [48]. Once there, it is unclear how the GEF activity of ELMO/DOCK180 is activated. Similarly, the PtdSer-binding protein BAI1 activates the GEF activity of ELMO/DOCK180, also through machanisms that are incompletely understood, but unlike the bridging molecule receptors, BAI1 interacts directly with ELMO/DOCK180 [30].

Post-engulfment, a host of pro-efferocytic and wound-healing signaling events are initiated and lead to immunosuppression, altered immunometabolism, M2-like macrophage polarization, and wound healing, profoundly impacting the resultant phenotype of the local tissue microenvironment (Fig. 1c). The nuclear neceptor (NR) family of transcriptional factors, specifically liver X receptor (LXR)-α and peroxisome proliferator-activated receptor (PPAR)-γ, become activated in response to efferocytosis, and play a key role in shaping the efferocytosis-induced anti-inflammatory phenotype [49]. However, the mechanism(s) by which LXRα and PPARγ become activated during efferocytosis have only recently been uncovered and are very much dependent upon trafficking and processing of the engulfed apoptotic cell within the phagosome. Intriginguely, components of the autophagy machinery have been identified on phagosomes containing apoptotic cells and are essential for apoptotic cell processing and subsequent immunomodulation. Specifically, LC3-associated phagocytosis (LAP), a process in which the hallmark protein of the autophagy machinery, LC3, is conjugated to the phagosome, provides a mechanistic link between engulfment of the dying cells and LXRα/PPARγ activation [50]. Although LAP uses machinery associated with autophagy, LAP is distinct from canonical and non-canonical forms of autophagy. There is a growing body of evidence from untransformed tissues showing that LAP is required for effective clearance of dying cells [51].

During LAP, the autophagy-related proteins LC3, Vsp34, Vsp15, Beclin, and UVRAG, but not the autophagy-related proteins ULK1 or Atg7, are recruited to the phagosomal surface rapidly after AC engulfment, generating what is known as a ‘LAP’osome [51]. The protein Rubicon, which largely inhibits autophagy, is also required during LAP [52]. LAPosome formation is necessary for phagosomal trafficking to, and fusion to, lysosomes, where the dying cell corpse is degraded by acidic proteases, lipases, and nucleases of the lysosome, and where cholesterols derived from the dying cell are either partitioned for efflux, or esterified then hydrolyzed by lysosomal acid lipase into 25-hydroxysterol and 27-hydroxysterol, the ligands responsible for activation of LXRα [49]. The digested fatty acids derived from the dying cell produce the ligands responsible for activation of PPARγ [53]. Together, ligand-activated LXRα/PPARγ activate the transcription of anti-inflammatory cytokine-encoding genes, including *Tgfb1*, *Il10*, and *Il13*. Moreover, LXRα and PPARγ upregulate transcription of genes encoding the efferocytic machinery, including those encoding MerTK, Tyro3, Axl, MFG-E8, Gas-6, and Rac1, while enhancing expression of lysosomal components, and genes encoding factors required to efficiently dispatch the engulfed and digested components, including the cholesterol efflux proteins ABCA1 and ABCG1 [54–56].

Similar to what is seen in mice defective for efferocytosis, mice with genetic aberrations that interefere with LAP are prone to uncontrolled inflammation and, in some cases, symptoms of autoimmunity. The importance of lysosomal degradation of the ingested cell corpse can be illustrated by observations of phagocytes lacking lysosomal DNAse II, which inefficiently degrade DNA from the engulfed corpse, thus allowing the accumulated cytoplasmic DNA fragments to activate cytoplasmic DNA sensors that trigger innate immunity and inflammation [57]. Failure to properly digest cholesterol within the lysosome leads to the precipitation of toxic cholesterol salts within cytoplasmic compartments of the phagocyte, decreasing phagocytic cell survival, and increasing inflammation [58]. Phagocytes deficient for lysosomal acid lipase fail to activate LXRα following efferocytosis, impairing expression of anti-inflammatory cytokines, and also decreasing expression of genes encoding the efferocytic machinery, cholesterol efflux pathways, and phagocytic cell survival [59]. Thus, LAP sits at the crossroads of efferocytosis and the resultant anti-inflammatory response.

Once the apoptotic cell is engulfed, the phagocyte now carries an excess of lipids, cholesterols, carbohydrates, proteins, and nucleic acids, dramatically altering the metabolic load of the phagocyte, and potentially contributing to mitochondrial oxidative stress, a highly pro-inflammatory event. The phagocyte adapts to the metabolic emergency created by efferocytosis in part by increasing Drp1-mediated mitochondrial fission at the mitochondrial associated membrane (MAM), thus allowing Ca^2+^ release into the cytoplasm, which has two important effects [60]. First, Ca^2+^ release lowers mitochondrial membrane potential, relieving mitochondrial oxidative stress. Second, Ca^2+^ aids in LAPosomal trafficking to and fusion with lysosomes. Phagocytes also increase mitochondrial biogenesis, increase fatty acid oxidation, and increase oxidative phosphorylation (OxPhos) to meet the shifting metabolic requirements of efferocytosis [49]. Interestingly, the capacity for a cell to engage and upregulate OxPhos and mitochondrial fission influences not only its survival, but also its future success as an efferocyte and its ability to produce an anti-inflammatory phenotype.

### Efferocytosis in the tumor microenvironment

**Fig. 2:**
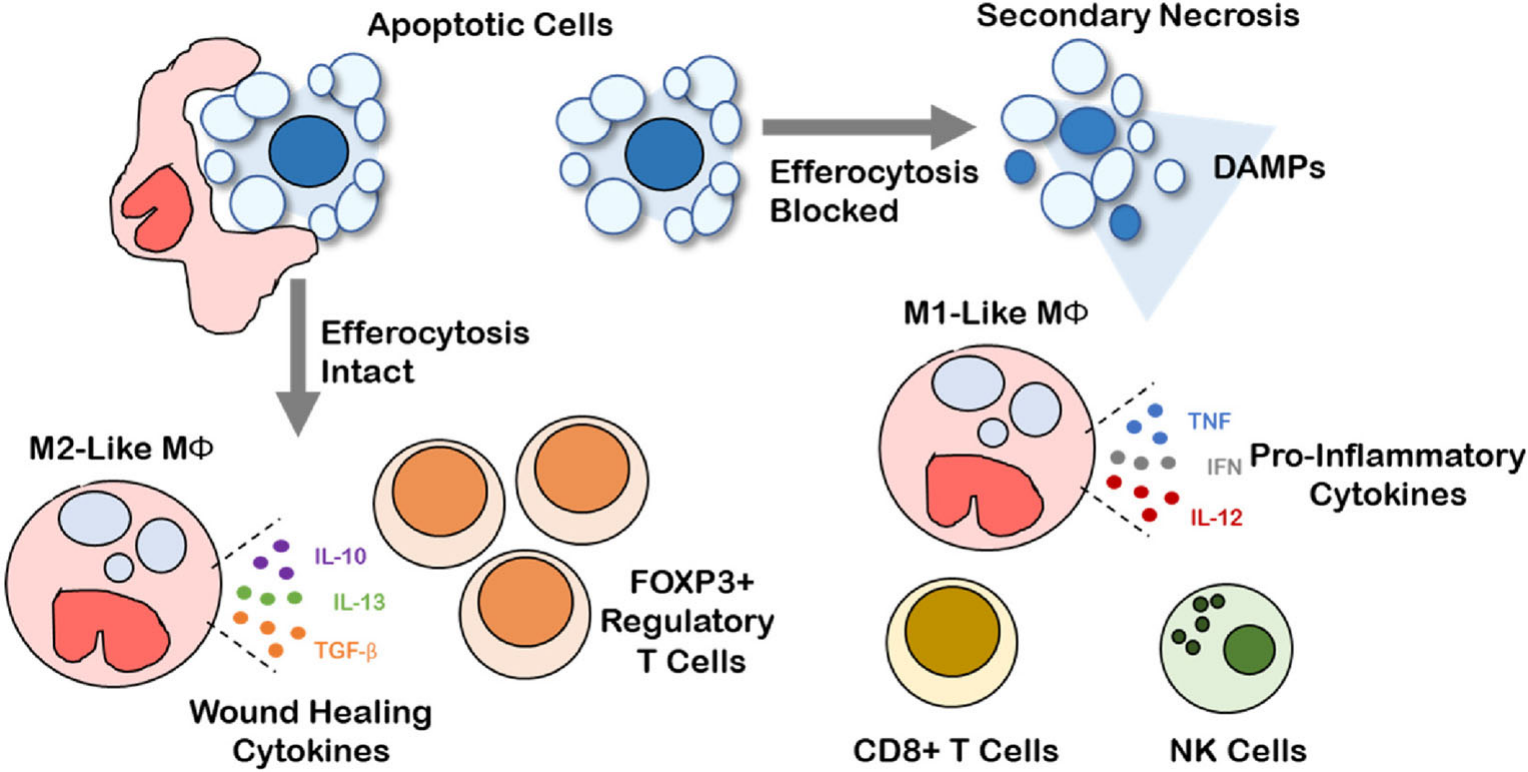
Efferocytosis shapes a pro-tolerogenic tumor microenvironment. Intact efferocytosis leads to efficient clearance of apoptotic cells in the tumor microenvironment. Efferocytosis leads to M2-like macrophage polarization, production of wound healing cytokines (e.g., IL-10, IL-13, TGF-β), and recruitment of FOXP3+ regulatory T cells, promoting a tolerogenic and immunosuppressive tumor microenvironment. Secondary necrosis occurs when efferocytosis is blocked leading to the release of pro-inflammatory damage-associated molecular patterns (DAMPs). Pro-inflammatory DAMPs drive M1-like macrophage polarization, production of pro-inflammatory cytokines (e.g., TNF, IFN, IL-12), and recruitment of cytotoxic cells such as CD8+ T cells and natural killer cells that mediate anti-tumor immunity

Cell death is a common event in solid tumors during malignant progression, and increases further with cytotoxic treatment. The type of cell death undergone (i.e., apoptosis, necrosis, necroptosis, or pyroptosis) and subsequent mechanisms of corpse clearance have a profound impact on the immune phenotype within the TME (Fig. 2). Clearance of dying cell corpses in the TME via efferocytosis is canonically an immunosuppressive phenomenon [61]. In contrast necrosis and secondary necrosis of uncleared apoptotic cells promotes a pro-inflammatory landscape and anti-tumor immunity. Interestingly, clearance of live tumor cells by phagocytosis may also serve as a pro-inflammatory process. For example, inhibition of the “do not eat-me” CD47-SIRPα interaction between tumor cells and phagocytic macrophages leads to an anti-tumor immune response characterized by the presence of CD8+ cytotoxic T lymphocytes and absence of FOXP3+ immunosuppressive T regulatory cells [62, 63].

Efferocytosis creates an immunosuppresive phenotype within tumors through a coordinated series of signaling events involving multiple compartments of the tumor milieu [61, 64]. Efferocytosis limits the presence of pro-inflammatory DAMPs released by secondary necrotic, uncleared corpses. Consequently, cytokine production is shifted from what might otherwise have been immunostimulatory (e.g., TNF, IFN, IL-12) to immunosuppressive (e.g., IL-4, IL-10, IL-13, and TGF-β) when efferocytosis is functional in the TME. During efferocytosis, tumor-associated macrophages (TAMs) are polarized toward an M2-like wound healing phenotype through metabolic signaling downstream of PPARγ and LXRα. Anti-inflammatory and inflammation-resolving cytokines produced upon efferocytosis recruit FOXP3+ regulatory T cells, which potently suppress the effector functions of CD4+ and CD8+ effector T cells within the TME. Further, efferocytosis upregulates MerTK, Axl, and Tyro3 expression and activity on tumor macrophages and DCs, promoting polarization towards an immunosuppressive phenotype. Because DCs and macrophages are important antigen presenting cells that lie at the interface of innate and adaptive immunity, the efferocytosis-mediated shift in DC and macrophage phenotypes result in decreased antigen cross presentation to T cells, decreased T cell clonal expansion, and impaired development of antigen-dependent anti-tumor immunity. Thus, the cumulative effect of efferocytosis serves to limit tumor inflammation and anti-tumor immunity and suggests that efferocytosis is an underappreciated immune checkpoint, perhaps one that might be therapeutically targeted in the setting of cancer.

Interestingly, results linking efferocytosis to cancer progression are increasing. For example, blockade of efferocytosis using Annexin V to block PtdSer from interacting with the efferocytosis machinery of phagocytes sufficiently reduces tumor progression and metastasis [65, 66]. Further, the efferocytosis receptor MerTK correlates with poor survival in numerous human cancers, as does its PtdSer bridging ligand Gas6 [64, 67–72]. Notably, α_v_β_3_ integrin and its PtdSer bridging ligand MFG-E8 are dysregulated in preclinical models of prosate and triple negative breast cancers [73, 74]. The PtdSer receptor TIM4 has garnered much attention as a potential cancer vaccine adjuvant [75–77]. Although a growing body of evidence supports the idea that efferocytosis in the tumor microenvironment increases tumor progression, it remains to be seen whether these molecules exhibit their pro-tumorogenic effect primarily through efferocytosis, per se, or if the pro-tumorigenic impact of each molecule is multi-faceted. For example, recent findings indicate roles for MerTK [78, 79], MFG-E8 [73], and PtdSer [65, 66] in the development and progression of neoplasia specific to their efferocytic functions. However, MFG-E8 and Gas6 are also known to increase macrophage polarization independently of efferocytosis [80, 81]. Thus, dissecting the impact of efferocytosis from other effects of Gas6 or MFG-E8 signaling might be difficult.

A series of studies implicated oncogenic roles for MerTK where its expression correlated with poor prognosis and chemoresistance in solid tumors and blood cancers [64, 67–69, 71, 72, 82]. Several studies show that tumor associated macrophages use MerTK signaling to produce a more agressive and tolerogenic tumor microenvironment. For example, tumors grew more slowly and were poorly metastatic when MerTK is absent from the leukocyte cellular compartment [78]. Importantly, transplantation of *MerTK*^*−/−*^ bone marrow into wild-type mice decreased tumor growth and altered cytokine production whereas transplantation of wild-type bone marrow had no such effects, strengthening the link to a leukocyte-specific role for the oncogenesis of MerTK. Interestingly, breast cancer progression is accelerated in the postpartum mammary gland, a microenvironment with widespread programmed cell death and high levels of efferocytosis [83–85]. Using both spontaneous and allografted mammary tumor models in fully immune-competent mice, it was shown that dying mouse mammary tumor cells, even those occurring in the context of post-lactational involution, are cleared through MerTK-dependent efferocytosis, which drives the robust induction of immunosuppressive cytokines IL-4, IL-10, IL-13, and TGF-β [79]. Moreover, genetic ablation or pharmacologic inhibition of MerTK in these models reduced M2-like macrophages, decreased wound-healing cytokine production, and blocked formation of postpartum tumor metastases. These studies strongly suggest that MerTK-mediated efferocytosis promotes a wound-healing microenvironment that drives metastatic tumor progression during post-partum involution of the breast.

### Therapeutic targeting of efferocytosis in the setting of cancer

The tolerogenic and anti-inflammatory impact of efferocytosis on the microenvironment of untransformed tissues is decidedly important to avoid tissue damage initiated by unrestrained inflammation. However, in the context of the tumor microenvironment, the anti-inflammatory phenotype generated by efferocytosis would be undesirable. Further, it is possible that the tolerogenic and anti-inflammatory phenotype generated by efferocytosis would be amplified under conditions in which tumor cell death was widespread, such as might be seen in response to cytotoxic, anti-cancer treatments. If all tumor cells were eliminated in response to cancer treatment, then the consequences of tumor cell apoptosis and efferocytosis would be a moot point. Instead, a significant proportion of solid tumors treated with targeted therapy, chemotherapy, or radiation do not exhibit pathological complete response (pCR) in the pre-surgical (neoadjuvant) setting, but rather exhibit partial response (PR) or stable disease (SD). Although in these cases of PR or SD the tumor is surgically excised following neoadjuvant treatment, lack of pCR is a strong predictor of tumor recurrence. Many molecular traits of tumor cells undoubtedly contribute to lack of pCR and the ensuing poor patient outcome, but it is critical to understand how efferocytosis might affect tumors following therapeutically induced tumor cell death, given that efferocytosis may endow immune tolerance to any tumor cells remaining in the post-neoadjuvant treatment setting.

PtdSer targeting shows efficacy in pre-clinical models of lung [86], breast [87], pancreatic [88], and brain tumors [89]. The anti-PtdSer antibody, Bavituximab, has been combined with current clinical standards-of-care in early phase II clinical trials for HER2-negative metastatic breast cancer and advanced non-small-cell lung cancer [90, 91]. In the pre-clinical studies, blockade of PtdSer using either Annexin V protein or anti-PtdSer mAb promoted anti-tumor immunity through induction of M1-macrophage polarization, increased dendritic cell maturation and antigen presentation, and increased presence of CD8+ cytotoxic T cells within the tumor microenvironment [86, 88, 89, 92]. As expected due to the role of PtdSer in efferocytosis, and the impact of efferocytosis on M2 macrophage polarization, anti-PtdSer antibodies also reduce M2-like tumor associated macrophages and alter cytokine expression profiles from immunosuppressive to immunostimulatory [92].

Several small molecular weight inhibitors have been developed that may have the potential to block efferocytosis in the cancer setting. These include the AXL inhibitor BGB324 (also known as R428), currently in a phase 1b study in erlotinib-sensitive and refractory patients with stage III and IV non-small cell lung cancer (NSCLC) [93], and the MerTK inhibitor UNC-2025 [94], which has been reported to block growth of melanoma, NSCLC [95], and other tumor models in preclinical studies [96]. However, these studies have been directed primarily at Axl- and MerTK-expressing cells within the tumor cell compartment, rather than in phagocytes of the tumor microenvironment, and clinical studies examining efferocytosis as an objective have not been reported. Pre-clinical studies using the MerTK inhibitor BMS-777607, a small molecule inhibitor that also targets the RTKs Met, Ron, Tyro3, and Axl [97], confirmed that efferocytosis can be blocked within highly apoptotic post-lactational mammary tumors [79]. Importantly, these studies confirmed that inhibition of efferocytosis was sufficient to halt tumor progression through blockage of efferocytosis-induced anti-inflammatory cytokines such as TGF-β and IL-10, and potentially through increased secondary necrosis and subsequent inflammation. Neutralizing antibodies that block TGF-β signaling confirmed the role of this immunosuppressive cytokine in efferocytosis-induced tumor progression in the setting of post-lactational involution of the mammary gland. A separate, elegant study confirmed the role of IL-10 in progression of mammary tumors in the setting of post-lactational involution [98]. In the context of therapeutically rendered tumor cell death, one study demonstrated that tumor-associated macrophages potently upregulate MerTK following radiation-induced tumor cell killing and that loss of *Mertk*, or inhibition of TGF-β (SM16) combined with radiation controlled post-radiation tumor progression to a greater extent that radiation alone [99]. Although these studies are pre-clinical, these findings support the idea that efferocytosis of apoptotic tumor cells favors tumor progression through production of anti-inflammatory and tolerogenic cytokines.

## Conclusion

Clearance of apoptotic cells prevents and resolves inflammation, at the same time increasing immune tolerance to antigens derived from the apoptotic cells. While these results are beneficial in the setting of normal physiology, and in most cases of inflammation, apoptotic cell clearance can have deleterious consequences within the tumor microenvironment, potentially affecting the natural progression of the disease, and perhaps thwarting much of the benefit derived from cancer treatments that induce tumor cell death. Our increasing knowledge of the molecular pathways used by phagocytes to engulf, traffic, and degrade apoptotic cells may reveal points of vulnerability for therapeutic targeting of efferocytosis. With this as the goal, it is possible that inhibition of efferocytosis in combination with cancer treatments might promote secondary necrosis in the tumor microenvironment, potentially increasing tumor-infiltrating lymphocytes capable of anti-tumor immuninty and improving the clinical responses to treatments across a variety of cancers.
